# 3D printing in orthodontics - Past, present and future: A systematic review and meta-analysis

**DOI:** 10.6026/973206300211766

**Published:** 2025-06-30

**Authors:** Sonia Dubey, Bhagabati Prasad Dash, Biswaroop Mohanty, Sanghamitra Jena, Nivedita Sahoo, Swati Singh

**Affiliations:** 1 Department of Orthodontics and Dentofacial Orthopaedics, Kalinga Institute of Dental Sciences, Bhubaneswar, Odisha, India

**Keywords:** 3D printing technology, orthodontics, CAD-CAM, three-dimensional, dentistry, digital technology, orthodontic appliances

## Abstract

The accuracy of orthodontic appliances made with 3D printing, such as aligners, bonding trays and surgical splints, compared to traditional methods is of interest. Fifteen studies were analyzed, focusing on fit, dimensional deviations and accuracy. Results showed 3D-printed aligners offer better accuracy and resistance than conventional ones. The fit of 3D-printed indirect bonding trays and retainers was clinically acceptable, with gypsum casts showing less volumetric change. 3D-printed NAM aligners improved accuracy, comfort and nasal symmetry. Thus, 3D printing offers better precision, accessibility and patient care in orthodontics.

## Background:

3D printing has emerged as a groundbreaking technology in orthodontics, reshaping traditional approaches to dental treatments through precision, efficiency and customization [1]. This additive manufacturing process creates three-dimensional objects layer by layer, starting from a digital model, making it especially valuable in crafting patient-specific orthodontic appliances [2]. Its applications are vast, ranging from the production of clear aligners and retainers to orthodontic brackets and wires tailored to individual treatment plans. It also plays a pivotal role in manufacturing surgical guides and splints used in complex procedures, as well as diagnostic models that aid in planning treatments and educating patients [3]. Furthermore, 3D printing facilitates the creation of expansion devices, such as palatal expanders, which enhance the scope of orthodontic care. One of the greatest advantages of 3D printing in orthodontics is its ability to customize appliances to fit the unique dental anatomy of each patient, resulting in improved treatment outcomes [4]. The efficiency of this technology reduces the time required for device fabrication, thereby streamlining the workflow in orthodontic practices. Additionally, the reduction in material waste and labour often makes 3D printing a cost-effective solution, benefiting both practitioners and patients. The use of digital impressions, coupled with the precision of 3D-printed appliances, greatly enhances patient comfort and satisfaction during treatment. The innovative design possibilities offered by 3D printing further allow practitioners to explore complex and multi-material devices that were previously unattainable [5]. Despite its promising advantages, 3D printing in orthodontics has its limitations. The materials used for printing often face challenges related to durability and biocompatibility, raising concerns about their long-term reliability [6]. The initial setup cost for 3D printing equipment and software can be significant, especially for smaller clinics and may deter widespread adoption. Moreover, the post-processing steps required for printed appliances, such as cleaning and curing, can be time-consuming. The specialized training and expertise needed to operate 3D printers effectively also pose a barrier to adoption. Regulatory compliance for medical devices is another hurdle, as ensuring adherence to these standards can be complex and demanding [7]. Therefore, it is of interest to describe the current state of 3D printing technology in orthodontics.

## Methods:

## Protocol and registration:

The PRISMA 2020 statement reporting standards for systematic reviews and meta-analyses were followed in the reporting in this review. This systematic review was registered under PROSPERO 2023 CRD42023462254 registration number and submitted to the PROSPERO database of the International Prospective Register of Systematic Reviews.

## Search strategy:

A comprehensive electronic literature search was conducted across multiple databases, including PubMed, Scopus and Google Scholar, to retrieve relevant articles on the study topic. The search strategy, including keywords and the number of papers identified in each database, is outlined in Table 1 (see PDF). Additionally, grey literature was explored using Open Grey and Grey Net International. To ensure accuracy and eliminate duplicate records, the duplicate removal tool in Rayyan. Ai 8 was employed.

## Screening and selection of studies:

The literature search was independently conducted by two investigators, following a stepwise approach. Initially, articles retrieved from the electronic search were screened based on their title and abstract. After eliminating duplicates, a manual reference search was performed to identify additional relevant studies that may have been overlooked in the initial search. The final selection of articles was determined through a full-text review, applying predefined inclusion and exclusion criteria. Any disagreements during the selection process were resolved through discussion and mutual consensus. Inclusion criteria were prospective or retrospective clinical trials, observational studies and *in vitro* studies. Exclusion criteria were reviews, authors' opinions, thesis articles, case reports and case series. Duplicate studies were eliminated after all the retrieved studies. Two authors independently selected the studies twice. Following the reading of abstracts and the elimination of non-eligible papers, possibly pertinent studies were identified by title. Following this step, a manual search of the eligible studies' references was conducted to uncover any undiscovered new papers. After thoroughly reading the articles, a decision was taken in accordance with the prerequisite.

## Study design:

Observational studies- case-control and cohort studies is shown in Table 2 (see PDF).

## Main outcome(s):

[1] Accuracy of the arch and tooth dimensions in printed models

[2] The precision of the degree of fit in surgical splints in orthognathic surgery

[3] Accuracy of printed aligners'/retainers' degree of fit

[4] accuracy of bracket tip, torque

[5] indirect tray bonding accuracy

## Measures of effect:

The precision of fit between acrylic surgical splints and 3D printed splints, as well as the mean difference in linear measurements between plaster/digital models and 3D printed models. Accuracy of expression of tip, torque of conventional bracket and 3 D printed bracket.

## Additional outcome(s):

Influencing factors of the accuracy of 3D printed aligners or retainers. Mechanical properties of 3D printed aligners or retainers.

Initially, 2624 articles were retrieved. After duplicate removal and title and abstract screening by the two authors, 48 articles remained for full text evaluation. Finally, 15 articles were included in the analysis (Figure 1 - see PDF) (Prisma flow chart selection of records). As shown in Table 4 (see PDF), the quality of the randomized controlled trials (RCTs) was assessed based on several methodological criteria. Similarly, Table 5 (see PDF) presents the quality assessment of non-randomized experimental studies, evaluating factors such as cause-effect clarity, treatment similarity and statistical analysis.

## Results:

Out of 15 studies, 2 studies evaluated the characteristics of direct printed aligner, 3 on indirect bonding tray, 2 on 3D printed models, 2 on 3D NAM [naso-alveolar molding], 2 on 3D printed splints and 4 studies on 3D printed retainers.

## Risk of bias assessment:

To assess the risk of bias, the QUADAS-2.0 tool for the prospective randomized clinical trial according to the Cochrane guidelines, were applied. Two review authors independently performed the risk of bias assessment. A third author resolved any disagreement (Table 3 - see PDF). Figures 2 and 3 (see PDF) shows, the risk of bias assessment for randomized and non-randomized experimental studies, respectively, while Figures 4 and 5 (see PDF) illustrates the risk of bias assessment for randomized and non-randomized experimental studies.

## Discussion:

3D-printed cured clear dental aligners demonstrated superior geometric accuracy, with an average relative difference in tooth height of 2.55% compared to 4.41% for thermoformed aligners. Tooth height measurements exhibited low standard deviations (0.03-0.09 mm) across all observers for both types of aligners. In terms of mechanical performance, 3D-printed aligners withstood a maximum load of approximately 662 N for a displacement of 2.93 mm, significantly surpassing thermoformed aligners, which endured only 105 N for the same displacement. Thermoformed aligners underwent irreversible plastic deformation at larger displacements, whereas 3D-printed aligners exhibited elastic deformation, allowing for recovery at lower displacements [23]. Similarly, Koenig *et al.* [9] evaluated three types of aligners developed using the same standard tessellation language (STL) file, including thermoformed aligners made from Zendura FLX^TM^ and Essix ACE^TM^, as well as direct-printed aligners using Tera Harz^TM^ TC-85DAP 3D Printer UV Resin. No digital tooth movement software was used in the process. To assess accuracy, the aligners were sprayed with opaque scanning spray, scanned and imported into Geomagic® Control X^TM^ metrology software, where they were superimposed onto the reference STL file using a best-fit alignment algorithm. Measurements taken at nine anatomical landmarks revealed varying levels of accuracy. Mean absolute discrepancies for Zendura FLX^TM^ ranged from 0.076 ± 0.057 mm to 0.260 ± 0.089 mm, Essix ACE^TM^ ranged from 0.188 ± 0.271 mm to 0.457 ± 0.350 mm and direct-printed aligners ranged from 0.079 ± 0.054 mm to 0.224 ± 0.041 mm. Root mean square (RMS) values, indicating overall trueness, were recorded as 0.209 ± 0.094 mm for Essix ACE^TM^, 0.188 ± 0.074 mm for Zendura FLX^TM^ and 0.140 ± 0.020 mm for direct-printed aligners. The findings suggest that direct-printed aligners exhibit greater trueness and precision compared to thermoformed aligners, reinforcing their potential as a more accurate orthodontic solution. Printed aligners offer a streamlined manufacturing process by directly exporting virtual models from CAD software to the printer's system, requiring only post-processing steps such as centrifugation, support removal and UV curing. CAD software facilitates the design of intricate geometries and enables efficient workflows, allowing rapid aligner delivery. Additionally, the software offers an option for variable thickness in strategic areas to enhance precision in tooth movement. For example, when moving a central incisor labially, thickness is increased on the palatal side, whereas for lateral incisor derotation, extra thickness is added to specific lingual and labial regions. However, further *in vitro* and *in vivo* studies are needed to validate the effectiveness of these thickness adjustments in optimizing aligner performance. Digital indirect bonding offers the benefits of traditional indirect bonding while streamlining the process through a fully digital workflow. It enables computer-aided bracket positioning, predictive treatment outcomes, standardised tray fabrication and reduces manufacturing steps, enhancing efficiency and precision in orthodontic treatment [24]. Bracket positioning accuracy in indirect bonding using 3D-printed trays was assessed across multiple dimensions by Bachour *et al.* [12]. Mean placement deviations were recorded as 0.10 mm for both mesiodistal and buccolingual measurements and 0.18 mm for occlusogingival measurements, with bracket positioning within the 0.5-mm threshold occurring in 96.4% to 100% of cases. Linear positioning errors remained within acceptable limits, demonstrating reliable transfer accuracy. However, angular discrepancies were observed, with mean deviations of 2.558 for torque, 2.018 for tip and 2.478 for rotation, yielding accuracy rates ranging from 46.0% to 57.0%. These angular errors exceeded the acceptable thresholds, potentially due to limitations in the scan data. Overall, indirect bonding using 3D-printed trays enables precise bracket placement in linear dimensions, but further refinement is needed to improve transfer accuracy for torque, tip and rotation.

Similarly Duarte *et al.* [10] evaluated digital indirect bonding using Ortho Analyser (3Shape) software performed on prototyped models with identical malocclusion, utilising 3D-printed transfer trays for Mini Sprint Roth and Bio Quick self-ligating brackets. Bracket positioning accuracy was assessed across vertical, horizontal and angulation dimensions. Reproducibility was confirmed through repeat measurements by three orthodontists, analysed via Bland-Altman plots and intraclass correlation coefficients. Statistical analysis showed no significant bracket positioning differences, except for minor mesiodistal discrepancies in the Bio Quick group (P = .016), which were not clinically relevant. Horizontal variations ranged from 0.04 to 0.13 mm, with angulation discrepancies between 0.458 and 2.038 degrees. Orthodontists' experience had no significant impact on accuracy (P = .314 and P = .158). The study validated the reliability of digital indirect bonding with 3D-printed transfer trays, ensuring consistent bracket placement across multiple orthodontists. Schwarzler *et al.* [11] randomized clinical trial confirmed the reliability of CAD/CAM technology in indirect bracket bonding. Both hard and soft resin exhibited low immediate bracket loss rates compared to existing studies. Soft resin demonstrated superior accuracy and usability over hard resin. However, molar bracket bonding proved significantly less precise than incisor bracket placement. Sim *et al.* [23] concluded digital models had smaller root mean square values of trueness of the complete arch and preparations than stone models. However, the accuracy of the complete arch and trueness of the preparations of 3D printed models were inferior to those of the other groups. Park *et al.* [14] found the volumetric variations between conventionally produced casts and those fabricated using 3D printing exhibited notable differences. Conventional casts demonstrated smaller volumetric changes compared to their 3D-printed counterparts. Among the various 3D printing technologies, statistically significant distinctions (P<.05) were observed. Notably, ultraviolet-polymerizing polymer utilizing digital light processing displayed the least volumetric change. Analysis through 3D color mapping revealed consistent deformation patterns across all 3D printing techniques. Jaber *et al.* [13] did a study aimed to evaluate the accuracy of physical reproductions of plaster orthodontic study casts, created using two distinct rapid prototyping techniques: Fused Deposition Modeling (FDM) and Digital Light Processing (DLP) and they found no statistically significant differences between the 3D-printed models and their corresponding plaster counterparts. The mean overall deviation measured -0.11 mm for FDM and 0.00 mm for DLP, with variations ranging from -0.49 mm to 0.17 mm in the FDM technique and -0.42 mm to 0.50 mm in the DLP approach and they concluded that "The accuracy of the 3D models produced by the DLP and FDM techniques was acceptable." Similar results were shown by Brown *et al.* [24]. Where - as camardella *et al.* [25] found digital models produced using the SLA 3D printer, featuring a horseshoe-shaped base derived from intraoral scans, exhibit clinically significant transverse contraction. Due to this limitation, they cannot fully substitute traditional plaster models obtained from alginate impressions for orthodontic diagnosis and treatment planning. Similar results shown by Wan Hassan *et al.* [26], Rebong *et al.* [27].

NAM appliances produced through 3D printing have the potential to enhance precision, shorten appointment duration and lower expenses related to digital fabrication techniques. The use of rapid prototyping techniques has the potential to streamline the fabrication process for NAM appliances, enabling care providers to produce a complete set for a single patient in one step with precise accuracy. This approach can significantly reduce the need for multiple follow-up visits during treatment. Studies have demonstrated that NAM appliances designed through CAD technology yield comparable clinical results and present a similar likelihood of hard and soft tissue complications when compared to conventional NAM devices. Compared to the control group, the D-NAM (3D-printed nasoalveolar molding) demonstrated successful outcomes, showing significant clinical and/or statistical improvements in all measured MADs (maxillary arch dimensions) at T2. It effectively facilitated the approximation of the maxillary segments, reduced the cleft gap and enhanced arch symmetry. These findings align with the results reported by Shen *et al.* [16] and Loeffelbein *et al.* [28]. The fully printed intraoral plate proved to be effective, yielding comparable results to previous non-printed trials. The newly introduced D-NAM technique offers a simplified approach to NAM appliances, facilitating the refinement of MADs prior to surgical lip repair. 3D-printed NAM aligners demonstrated remarkable accuracy in fit and comfort, significantly improving pre-surgical alignment and nasal symmetry, while reducing the number of required visits. The fit of 3D-printed retainers closely matched vacuum-formed retainers. Cole *et al.* [19] concluded that traditional vacuum-formed retainers exhibited the smallest deviation from the original reference models, demonstrating greater accuracy in replication. In contrast, 3D-printed retainers showed the highest deviation at reference points on the smooth surfaces of the teeth while achieving close adaptation at the incisal edges and cusp tips. Overall, the fit of 3D-printed retainers appears comparable to that of vacuum-formed retainers, though additional clinical trials are necessary to evaluate their performance in practical applications. Williams *et al.* [29] in particular," concluded that the smooth facial surfaces of central incisors provided greater differences up to 0.480 mm." Naeem *et al.* [22] conducted a comparative analysis of the accuracy of fifteen 3D-printed clear retainers produced using four widely utilized 3D printing technologies: stereolithography (SLA), digital light processing (DLP), continuous light processing (cDLP) and polyjet photopolymer (PPP) printing. To assess precision, six reference points were examined, along with intercanine (ICW) and intermolar width (IMW), by digitally superimposing 3D models with the printed retainers. The study established an accuracy threshold of 0.25 mm, within which all four printing methods remained. Among them, PPP retainers demonstrated the smallest deviations in the incisor region, DLP in the canine region and both cDLP and SLA in the molar region. Regarding ICW and IMW measurements, PPP printing yielded the highest replication accuracy, followed by SLA, while DLP and cDLP exhibited greater discrepancies in width. The superior inter-arch consistency of PPP printing may be attributed to its horizontal printing orientation, in contrast to the angled printing approach of the other technologies, or its minimized printing height. Overall, the study determined that PPP and SLA printers delivered the highest accuracy, whereas DLP and cDLP were the most precise methods for fabricating retainers. "Retainers fabricated by SLA, DLP, continuous DLP and PPP technologies were shown to be clinically acceptable and accurate compared to the standard reference file. Based on both high precision and trueness, SLA and PPP printers yielded the most accurate retainers" concluded by Naeem *et al.* [22]. According to Aksakalli *et al.* [30] the tensile bond strength exhibited by 3D printed retainers is favorable for their clinical orthodontic use. Regardless of the printing technology employed, virtual planning for orthognathic surgery and the fabrication of splints using STL files of patients' occlusion have been found to be sufficiently accurate for surgical applications, effectively replacing traditional acrylic splints. The discrepancy between conventional model occlusion and virtual occlusion ranged between 0.45 mm and 0.6 mm, which falls within the acceptable 1.5 mm threshold for positional variation between the virtual intermaxillary position and the intraoperative intermediate intermaxillary relationship, as defined by Hernández-Alfaro [31]. Shqaidef *et al.* [32] reported no significant differences between printed and acrylic splints. Hanafy *et al.* [33] concluded that the implementation of CAD/CAM technology for patient-specific osteosynthesis and surgical guides has demonstrated a high degree of accuracy in translating virtual simulations into actual surgical procedures. This advancement has the potential to redefine the approach to maxillary positioning in standard clinical practice. However, despite its benefits, the primary drawback of the computer-assisted workflow remains its high cost. Similar results are shown by Kuehle *et al.* [34].

## Conclusion:

The transformative impact of 3D printing in orthodontics, emphasizing its precision, customization and efficiency is highlighted. Despite challenges such as material durability and high initial costs, the technology has the potential to significantly improve orthodontic outcomes and patient satisfaction. Further research and technological advancements will likely address current limitations, enhancing the clinical applicability and accessibility of 3D-printed orthodontic appliances.
